# CONcurrent ChEmotherapy and RadioTherapy in adjuvant treatment of breast cancer (CONCERT): a phase 2 study

**DOI:** 10.3332/ecancer.2023.1510

**Published:** 2023-02-23

**Authors:** Tabassum Wadasadawala, Akanksha Anup, Johnny Carlton, Rajiv Sarin, Sudeep Gupta, Vani Parmar, Rima Pathak, Jaya Ghosh, Jyoti Bajpai, Seema Gulia, Revathy Krishnamurthy

**Affiliations:** 1 Department of Radiation Oncology, Tata Memorial Hospital, Homi Bhabha National Institute, Mumbai 400012, India; 2 Department of Medical Oncology, Tata Memorial Hospital, Homi Bhabha National Institute, Mumbai 400012, India; 3 Department of Surgical Oncology, Tata Memorial Hospital, Homi Bhabha National Institute, Mumbai 400012, India

**Keywords:** concurrent, chemoradiation, taxanes, breast cancer

## Abstract

**Purpose:**

This phase 2 study evaluated the safety of adjuvant chemoradiation (CTRT) for breast cancer.

**Methods:**

From April 2019 to 2020, 60 patients with stage II–III invasive breast cancer planned for adjuvant taxane-based chemotherapy and radiotherapy (RT) were accrued. Local ± regional (excluding the internal mammary nodal region) RT (40 Gy in 15 fractions ± boost) was started with the third cycle of an adjuvant taxane in a 3-weekly schedule or with the eighth cycle in a weekly schedule.

**Results:**

Thirty-six patients received 3-weekly paclitaxel regimen and 24 received weekly paclitaxel regimen. The commonly used technique was three-dimensional conformal RT which was employed in 58% of patients. Regional RT, including the medial supraclavicular region, was done in 42 patients (70%). No dose-limiting (grade 3 or 4) toxicity was documented and all patients completed CTRT without any treatment interruption. The median ejection fraction pre and post CTRT 6 months was 60% (*p* = 0.177). The median value of cardiac enzyme (Troponin T ng/L) decreased from 37 to 20 (*p* = 0.009) post CTRT 6 months. Of the 54 patients who underwent the pulmonary function tests, there was no significant difference in various parameters like functional vital capacity (FVC) (2.29 versus 2.2 L, *p* = 0.375), forced expiratory volume at 1 second (FEV1) (1.86; 1.82; *p* = 0.365), FEV1/FVC (81.5; 81.43; *p* = 0.9) and diffusion lung capacity for carbon monoxide (88.3; 87.6; *p* = 0.62). At a median follow-up of 34 months, the 3-year actuarial rate of disease-free survival and overall survival was 75% and 98.3%, respectively. Quality of life scores (QOL) improved after treatment for most of the domains comparable to the pre-RT scores.

**Conclusion:**

Taxane-based adjuvant CTRT is a safe option and results in minimal toxicity and excellent compliance. It has favourable impact on cardio-pulmonary profile and QOL scores.

## Highlights

- Taxane-based adjuvant chemoradiation (CTRT) is a safe option and results in minimal toxicity and excellent compliance.
- It has favourable impact on cardio-pulmonary profile and quality of life (QOL) scores.

## Introduction

Breast cancer is the most common cancer in women worldwide, with 1.67 million new cases and 0.5 million deaths annually [1]. Large majority present with locally advanced disease in the low-middle-income countries like India [2]. Surgical extirpation followed by adjuvant radiotherapy (RT) to the primary and regional lymphatics is the standard of care to improve locoregional control and overall survival (OS). Anthracycline-based chemotherapy (CT) with addition of taxanes for patients with adverse factors is recommended as the first-line systemic therapy in order to reduce the risk of recurrence. Locally advanced breast cancers (LABCs) are at higher risk of locoregional failure; hence, it is undesirable to have undue delays in the overall treatment of breast cancer. However, as the competing risk of distant failure is higher than local failure, generally CT is either completed as neoadjuvant therapy or given as adjuvant treatment before RT [3]. With the prolongation of the CT due to increased number of cycles for LABCs, initiation of adjuvant RT is delayed typically by 6–8 months as it is offered towards the end of the treatment phase. The impact of such delayed initiation of adjuvant RT is unknown. Moreover, the waiting times for RT can potentially add to this undesired delay.

Concurrent CTRT has long been an attractive option to reduce delays in RT and improve local tumour control. Chemoradiation is a standard of care in many cancer sites like head-neck, lung, cervical, etc., both in the pre-operative and post-operative setting [4]. Though this has been attempted in breast cancer as well, there is a lack of randomised data on sequencing for CT and RT that can be applicable for modern treatment protocols. Concerns regarding increased rate of cardiopulmonary toxicities prevented large-scale adoption of this approach especially with anthracycline-based regimens. The concomitant use of doxorubicin or epirubicin produced 30%–44% rate of high-grade (grade 3 and 4) radiation dermatitis leading to treatment discontinuation. As the treatment protocols have changed over last few decades, it is important to test CTRT in the purview of modern CT (anthracycline/taxane based) and RT (hypofractionated) protocols. The advent of taxanes in CT regimens for node positive patients has rekindled the interest in concurrent CTRT. While being much less cardiotoxic than anthracyclines, taxanes also have a potent radio sensitising effect through cell cycle arrest at the G2-M junction. In this study, we evaluated the feasibility of concurrent adjuvant CTRT in breast cancer patients planned for taxane-based CT in the postoperative period.

## Materials and methods

### Patient population

The study was approved by the Institutional Ethical Committee (Project number 3155) and registered on clinical trial registry of India (CTRI/2019/02/017411). Written informed consent was obtained from eligible patients. This prospective interventional phase 2 study included patients with pathologically confirmed stage II–III invasive breast cancer, planned for taxane-based adjuvant CT and adjuvant RT after oncological resection. The exclusion criteria included patients with hypersensitivity to taxanes, extended-field RT (e.g. plan to target internal mammary nodes and/or axillary nodes, synchronous bilateral primary, unfavourable anatomy, etc.), undergone immediate whole breast reconstruction and who had received prior irradiation of breast/mediastinum/neck.

### Pre-treatment evaluation

At the time of presentation, all patients underwent assessment of routine blood parameters, bilateral mammography, biopsy of the tumour for histopathological confirmation with assessment of hormone receptor status for oestrogen receptor (ER), progesterone receptor (PR) and human epidermal growth factor receptor 2 (HER2). An appropriate imaging for the chest and abdomen was also done as per institutional protocol. Cardiac function evaluation was done with electrocardiogram (ECG) and 2D-echocardiography (2D-ECHO).

### Treatment details

Breast surgery constituted either modified radical mastectomy (MRM) or breast conservation surgery (BCS). Use of neoadjuvant CT was decided in multi-disciplinary tumour board, based on standard evidence-based guidelines and pre-treatment patient evaluation. Anthracyclines were given either in neoadjuvant setting or adjuvant setting as per standard institutional criteria. Adjuvant taxane CT was either 4 cycles of 3-weekly paclitaxel @ 175 mg/m^2^ or 12 cycles of weekly paclitaxel @ 75 mg/m^2^ which was given sequentially following anthracyclines. All HER2 positive patients received either weekly or 3-weekly adjuvant trastuzumab concurrent with the taxanes followed by 3-weekly maintenance therapy. Standard endocrine therapy for ER/PR positive tumours was recommended.

All patients received moderately hypofractionated locoregional RT as appropriate for the stage of cancer in line with the institutional protocols. The only difference was in the timing of delivering RT which was started with the third cycle of taxanes in a three-weekly schedule, or with the eighth cycle of taxanes in a weekly schedule. All patients underwent CT-based simulation and planning. Treatment was delivered using three-dimensional conformal RT (3DCRT) by a pair of tangential fields to encompass the breast/chest wall. For the irradiation of the supraclavicular fossa (SCF), a single direct anterior field covering the medial SCF was used. Mono-isocentric technique was used to avoid match line inhomogeneity. Forward or inverse planned intensity modulated RT (IMRT) was used as deemed necessary. Megavoltage photons (6–10 MV on linear accelerator) were used and cardiac shielding was used for all left-side cancers. A 5 mm bolus was used in all cases of mastectomy. The dose/fractionation regime used was 40 Gy in 15 fractions, 2.67 Gy per fraction given daily for 5 days a week. For patients who underwent BCS, a tumour bed boost was planned to a dose of 12.5 Gy in five fractions over 1 week sequentially in majority of the cases. Six patients (10%) received IMRT with simultaneous integrated boost. RT was started on the same day as CT.

### Follow-up protocol

All patients underwent pre-RT evaluation of cardiac and pulmonary function and filled the EORTC QLQ C30 and BR 23 questionnaires. During RT, they underwent weekly review and at conclusion they underwent acute toxicity assessment along with QOL assessment. A repeat assessment of cardio-pulmonary function and QOL was done at the first follow-up after 6 months. This was done for all patients irrespective of laterality. The assessment schedule is shown in Table 1.

### Statistical analysis

A convenience sample size of 60 patients was proposed. The toxicities attributed to concurrent therapy and occurring up to 6 months after the end of radiation therapy were recorded. Dose limiting toxicity (DLT) was defined as: any non-haematologic, non-cutaneous, non-pulmonary grade 3 or 4 toxicity; grade 4 granulocytopenia (absolute neutrophil count (ANC) < 500/mm^3^) for more than 7 days despite use of granulocyte colony-stimulating factor (G-CSF); any episode of febrile neutropenia (temperature < 38°C, ANC < 500/mm^3^) despite use of G-CSF; clinical sepsis in the setting of neutropenia; any grade 4 thrombocytopenia for more than 7 days; any delay of radiation therapy or treatment with paclitaxel for more than 2 weeks because of treatment-related side effects; any grade 4 skin toxicity using the Radiation Therapy Oncology Group/European Organization for Research and Treatment of Cancer Acute Radiation Morbidity scale (grade 4 included skin ulceration or necrosis, which involved one-third or more of the breast); any grade 3 lung toxicity using Radiation Therapy Oncology Group/European Organization for Research and Treatment of Cancer Late Radiation Morbidity scale (defined as low-grade fevers, cough and radiographic infiltrates) that required steroid therapy. The difference in compliance rate for the two regimens of taxanes (weekly and 3-weekly) was recorded as numbers and percentages and analysed using chi square test. The different grades of toxicity were assessed using Mann–Whitney *U* test. Continuous outcomes were analysed using unpaired *t*-tests. Survival outcomes were studied using Kaplan–Meier Curves in accordance with the DATECAN guidelines [5, 6]. All tests were performed at the two-sided 0.05 level. Analysis was carried out in Statistical Package for the Social Sciences Version 21.

## Results

### Patient demographics

Sixty patients were analysed. The demographic characteristics are depicted in Table 2. Tumours had invasive ductal histology with high histological grade (91.6%). Thirty-four patients (57%) were both ER and PR positive. Fifty-nine patients (98%) received all four cycles of anthracycline-based CT and only one patient could not complete the same due to compromised cardiac function. In this patient, the cardiac function recovered within 5 weeks and hence she tolerated adjuvant taxanes along with RT. Thirty-six patients received 3-weekly paclitaxel regimen and 24 patients received weekly paclitaxel regimen as depicted in Table 3. Of the 60 patients analysed, 35 patients (58.3%) were planned with 3DCRT and 25 patients (41.67%) were treated with IMRT. Supraclavicular irradiation was done in 50 (83.33%) of patients as shown in Table 3.

### Toxicity and compliance

**Figure 1.**
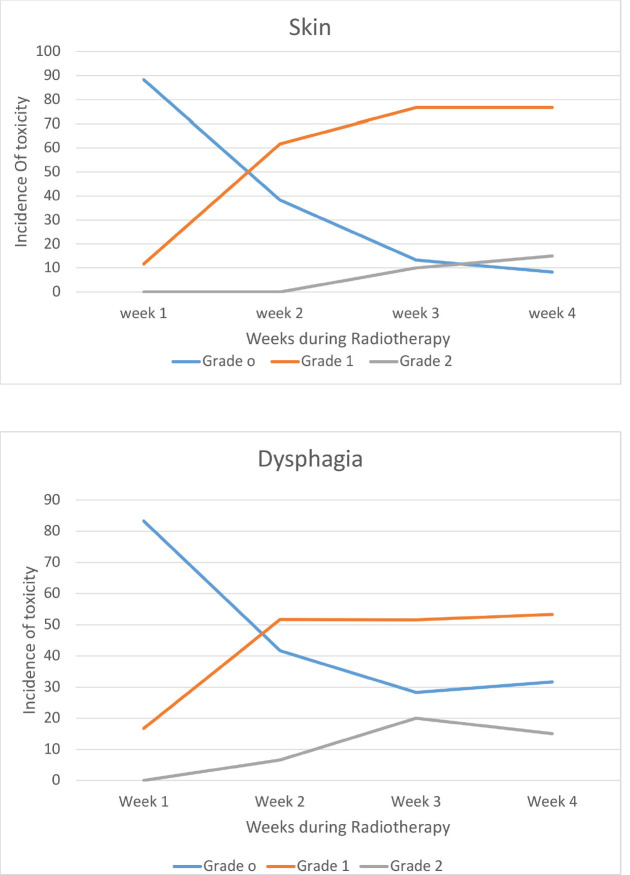
Toxicity – during chemoradiotherapy.

**Figure 2.**
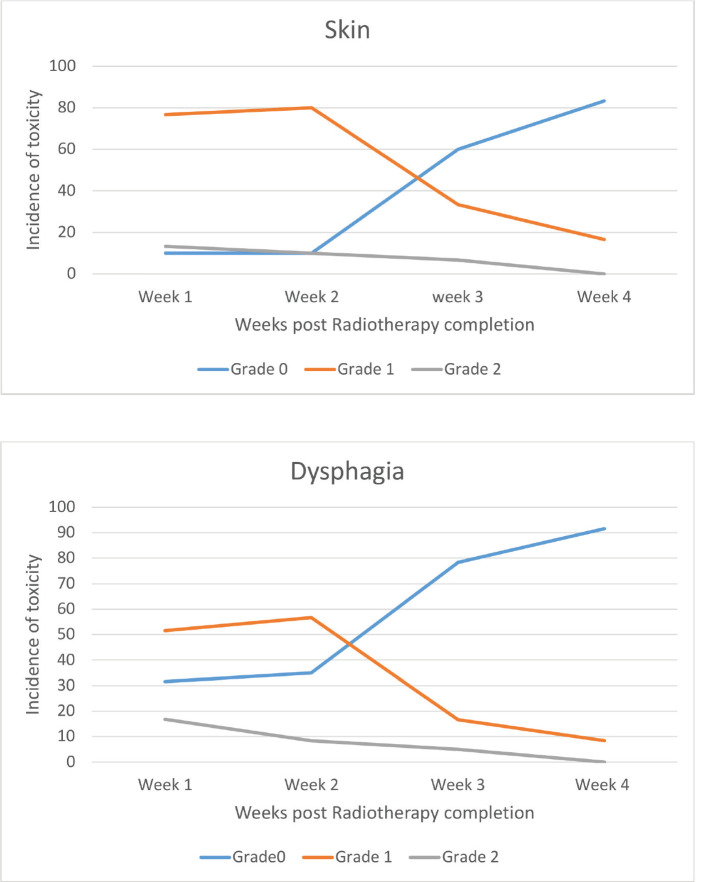
Toxicity – post chemoradiotherapy.

All the patients were compliant with taxane-based CT with dose reduction required only in three patients in view of peripheral neuropathy. All patients completed adjuvant RT without any treatment interruption either for taxanes or RT. None of the patients experienced DLT. Minimal CT-related toxicities were documented in the form of grade I fatigue in seven patients (11.6%), grade I peripheral neuropathy in eight patients (13.3%), grade 1 mucositis in five patients (8.3%) and three patients had grade 3 peripheral neuropathy for which they underwent paclitaxel dose reduction. No dose reduction was required for any other patient. RT-related toxicity was also minimal. The incidence of grade 0, 1 and 2 dermatitis was 4%, 78% and 12%, respectively, while grade 0, 1 and 2 dysphagia was 31%, 52% and 12%, respectively (Figure 1). There was no grade 3 toxicity. There was no difference between weekly and 3-weekly regimens with respect to compliance rate or toxicity. Dermatitis as well as dysphagia subsided completely by 4 weeks post-completion of CTRT (Figure 2). The median duration between first and last cycle of taxanes was 74 days (range: 62–107 days) and the median duration of RT start and conclusion was 23 days (range: 19–28 days). 48 patients (80%) had grade I lymphoedema and 12 patients (12%) had grade II The median time of occurrence of lymphoedema was 14 months. The dosimetric parameters for both IMRT and 3DCRT plans are shown in Table 4.

### Cardio-pulmonary function

**Figure 3.**
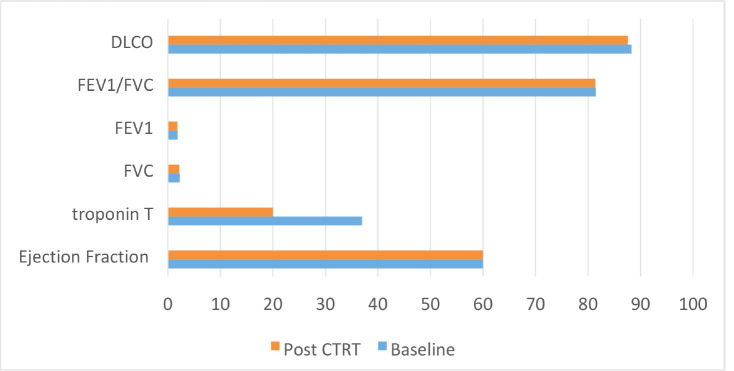
Bar chart showing the cardiopulmonary toxicity.

The median ejection fraction pre and post CTRT was 60% (*p* = 0.177). The median value of Troponin T at baseline was 37 which decreased to 20 post CTRT (*p* = 0.009) (normal range < 14 ng/L). Of the 54 patients who underwent pulmonary function test (PFT), the functional vital capacity (FVC) was 2.29 L at baseline which was 2.2 L post CTRT (*p* = 0.375). Similarly, no significant difference was seen with forced expiratory volume at 1 second (FEV1) (1.86; 1.82; *p* = 0.365), FEV1/FVC (81.5; 81.43; *p* = 0.9) and diffusion lung capacity for carbon monoxide (DLCO) at baseline and post CTRT (88.3; 87.6; *p* = 0.62), respectively. Figure 3 demonstrates the cardio-pulmonary profile of the study cohort.

### Quality of life

There was a significant improvement in QOL scores for nausea, vomiting, pain, constipation, loss of appetite and hair loss 6 months post RT conclusion. Table 5 reports the global QOL scores along with the breast-specific domains for QLQ-30 and BR-23.

### Disease-related outcome

Four patients had recurrence, one with out-of-field nodal recurrence in the retrosternal region (internal mammary nodal recurrence) and three patients developed distant recurrence only. At the median follow-up of 34 months, median disease-free survival (DFS) of 17.5 months (9–22 months) and median OS of 26 months (11–33 months). The 3-year actuarial rate of DFS and OS was 75% and 98.3%, respectively.

## Discussion

Adjuvant RT usually forms the terminal therapy in the multidisciplinary management of invasive breast cancer. Retrospective studies in the literature have reported high local recurrence rate when RT was delivered sequentially with CT In a study by Hartsell *et al* [7], it was seen that a delay in RT leads to increased risk of relapse in breast cancer; therefore, it was inferred that radiation should be delivered within 120 days after breast surgery. Long waitlist and access to RT in well-equipped facility are common barriers in the timely delivery of cancer care. This results in prolonged stay close to the cancer facility and subsequent increase in the out-of-pocket expenditure. Moreover, the usual norm of starting adjuvant RT 2–4 weeks after completion of CT defines a narrow interval for scheduling patients which may not ensure timely initiation of the treatment [8].

Three randomised phase III trials have been conducted which compared sequential CT followed by RT versus concomitant chemoradiotherapy [9–11]. In the ACROSEIN trial, 716 early breast cancer patients post breast conservation were randomised into 2 groups for concomitant or sequential administration along with the FNC protocol (5-fluorouracil 500 mg/m^2^, mitoxantrone 12 mg/m^2^ and cyclophosphamide 500 mg/m^2^). No significant difference in both arms was seen for 5-year DFS, locoregional failure free survival (LRFS), metastatic free survival and OS. The risk of loco-regional recurrence was reduced by 39% for node positive patients with CTRT. In the second trial by Arcangeli *et al* [10] with early breast cancer patients post breast conservation, CTRT was tested with cyclophosphamide, methotrexate and fluorouracil regimen. This trial also reported no difference in the 5-year oncological outcome with a 10-year local recurrence rate of 5%. In the third trial by Rouessé *et al* [11], 638 patients were randomised but fluorouracil, epirubicin and cyclophosphamide regime was tested concurrent with RT. There was no difference in OS or DFS in both the groups but it demonstrated a significant benefit in locoregional free survival for node positive patients. Patients receiving epirubicin had grade 3 and 4 nausea vomiting, grade 2–3 alopecia but a lower rate of cardiotoxicity (2% versus 6%, *p* = 0.02) and radiation dermatitis in the sequential RT arm (17% versus 24%, *p* = 0.03). All these three trials had used non-taxane protocols because these were standard during earlier times. Moreover, conventional fractionation was employed in all the trials.

The objective of the study was to evaluate the feasibility of concurrent taxane-based chemoradiotherapy in adjuvant setting for breast cancer. The rationale of conducting this type of study in the adjuvant setting was three-fold: reduction in the overall treatment time, potential radio-sensitisation and consequent improvement in the disease-related outcome. This phase 2 study has established the safety of adjuvant concurrent chemoradiation with taxanes. Hence, it will form the basis of phase 3 randomised trial that is planned to be carried in our centre with multi-centre collaboration. The current study is different from the available literature as CTRT was given with both weekly and 3-weekly paclitaxel-based regimens concurrent with moderately hypofractionated RT delivered using 3-D conformal technique or IMRT technique.

We did not want to restrict the eligibility criteria for any particular cohort as the idea was to test this regimen in all comers irrespective of laterality, type of surgery or prior exposure to anthracyclines. Forty-eight patients (80%) had received anthracycline-based neoadjuvant CT while 12 patients (20%) underwent upfront surgery. The cohort comprised of nearly equal number of right and left sided tumours as well as patients with BCS and MRM.

The trial from Massachusetts General Hospital had reported that the use of taxanes with RT either sequentially or concurrently can result in an increased rate of grade 2 or 3 pneumonitis. Their rate of symptomatic pneumonitis was 15% for patients receiving taxanes as compared to 1% for not receiving taxanes [12]. In this study, all the patients had received locoregional RT to the breast/chest wall and regional lymphatics. Supraclavicular–axillary lymph node areas were also included in 30 patients (equal in each group) while internal mammary lymph nodes were treated in 16 patients (10 in the group receiving sequential therapy and 6 in the group receiving concurrent therapy) through an independent *en face* electron field and in 25 patients (10 in sequential and 15 in concurrent groups) through a wide tangential field. The doses used ranged between 40 and 50 Gy, with a mean of 45 Gy and a boost to the tumour bed in lumpectomy patients varying between 16 and 20 Gy. The regional lymph node areas received a total dose ranging from 43.2 to 56.4 Gy, with a mean of 48 Gy. On the contrary, in our cohort, no incidence of symptomatic pneumonitis has been documented as we had not irradiated internal mammary nodes and also employed CT-based planning to deliver conformal RT.

Other authors have also similarly reported increased incidence of radiation pneumonitis [13–17] with concurrent use of paclitaxel and RT. In the study by Burstein *et al* [15], two of the seven patients receiving concurrent RT had dose limiting grade 3 radiation pneumonitis. Patients developed breathlessness and cough post RT conclusion with radiological evidence of ground glass opacities in the radiation field. To minimise the risk of pneumonitis, the patients received weekly paclitaxel on Friday to minimise overlap with radiation, which was not given on weekends. Patients were required to have simulation done by CT planning and were excluded from the protocol if the irradiated lung volume exceeded 18% of the ipsilateral lung, an arbitrary percentage thought large enough to allow radiation treatment in the majority of patients without excessive pulmonary exposure. However, in our study, the regimen was found to be pulmonary-safe both clinically and functionally. There was no symptomatic radiation pneumonitis leading to DLT. Neither did we exclude any patient based on any clinical or dosimetric parameter.

In the literature, studies have shown that paclitaxel infused twice weekly instead of weekly regimen leads to a significant reduction in toxicity [18]. However, in our study, it was seen that there was no significant difference in toxicity between 3-weekly and weekly paclitaxel regimen either during RT or even 1 month post completion of RT. The choice of the regimen was left to the treating medical oncologist to avoid any interference in the routine care. Both the regimens are well tolerated and completed without interruption.

Our study reports the 3-year OS of 98.3% and DFS of 75% at a median follow-up of 34 months for the entire cohort. There was no difference in OS in node positive and negative cohorts. In a recent small randomised trial by Lu *et al* [19] the 3-year DFS and OS were reported to be 76.9% and 64.9% for node positive, and 87.2% and 81.8% for node negative cohorts respectively, but without significant difference. They treated all patients with IMRT and reported no grade 3–4 radiation toxicity. The occurrence of grade 1–2 radiation skin reactions was similar in the weekly and 3-weekly paclitaxel groups (89.7% and 88.3%, *p* = 0.803). It also showed cardio-pulmonary safety [18, 19].

Cardiac troponin T is a well-established biomarker of cardiac damage in myocardial infarction due to ischaemic heart disease. In cancer patients, elevated troponin T and I levels have been reported after various CT regimens [20]. The baseline value of Troponin T in women of age more than 55 years and less than 75 years has been shown to be <4 ng/mL. Kitayama *et al* [21] reported that Troponin-T is useful in detecting preclinical myocardial damage due to anthracycline and trastuzumab. Furthermore, cardiac troponin is a deviation enzyme, and it is more useful for studying temporal changes at multiple time points rather than single one-time assessment. High-sensitive cardiac troponin assays have two advantages that can be helpful for monitoring and preventive strategies of CT-induced cardiotoxicity. First, myocardial damage can be evaluated multiple times with high-sensitive cardiac troponin assays which are also cost effective compared to 2D-ECHO cardiac magnetic resonance imaging and radionuclide angiography [21].

In low-middle-income countries where the out-of-pocket expenditure on cancer treatment outweighs the direct cost, such a simple approach of sequencing the adjuvant treatment, i.e., CT and RT is likely to have clinically and statistically significant health economic benefits. This is particularly relevant in low middle income countries (LMICs) where large majority of patients need taxane-based CT as well as adjuvant RT due to locally advanced stage at presentation.

## Conclusion

Concurrent adjuvant chemoradiotherapy in breast cancer has an acceptable toxicity profile. This shortens the overall treatment time and thereby has a great potential of being a cost-effective approach. The impact on disease outcome, QOL and health economic benefits needs to be studied in a randomised setting.

## Conflicts of interest

The authors declare no conflicts of interest.

## Funding statement

This study was funded through the intramural grant received from Tata Memorial Centre and Women’s Cancer Initiative (WCI).

## Figures and Tables

**Table 1.** Assessment time lines at every follow-up.

	Before RT	RT completion	Treatment completion	6th month	12th month	18th month	24th month
Clinical examination	√	√	√	√	√	√	√
Acute toxicity grading		√	√				
Late toxicity grading				√	√	√	√
Quality of life	√	√	√	√			
2D ECHO	√			√			
ECG	√			√			
MMG	√			√	√		√
PFT/DLCO	√			√			
Serum Troponin-T	√			√			

2D–ECHO, 2-Dimensional echocardiography; ECG, Electrocardiogram; MMG, Mammography; PFT/DLCO, Pulmonary function test/diffusion lung capacity for carbon monoxide

**Table 2.** Demographic characteristics.

Variables		Patients	
Age		Median 47 years (28–66)	
Menopausal status	Premenopausal	32	53.3%
	Perimenopausal	6	10%
	Postmenopausal	22	36.7%
Comorbidities	Diabetes mellitus	6	10.7%
	Hypertension	11	19.6%
	Tuberculosis	1	1.8%
	Thyroid	3	5.4%
Addictions	None		
Previous malignancy		4	7.1%
Laterality	Right	32	53.3%
	Left	28	46.7%
cT	1	14	23.4%
	2	30	50%
	3	6	10%
	4	10	16.6%￼
cN	0	10	16.66%
	1	30	50%
	2a	10	16.66%
	3a	10	16.7%
cM	0	54	90%
	1	6	10%
Histology	IDC	55	91.6%
	ILC	5	8.4%
Grade	I	3	5%
	II	9	15%
	III	48	80%
ER	Positive	37	63%
	Negative	23	37%
PR	Positive	35	59%
	Negative	25	41%
HER 2	Positive	28	46.7%
	Negative	32	53.3%
FISH	Amplified	9	15%
	Non amplified	13	21.6%
	Not done	38	63.3%

**Table 3.** Treatment characteristics of the cohort.

Type of surgery	
Breast conservation surgery	25 patients (41.66%)
Modified radical mastectomy	35 patients (58.3%)
RT type and target volume	
3DCRT IMRT	35 patients (58.3%) 25 patients (41.67%)
Breast + TBB Breast + TBB + SCF CW + SCF	10 patients (16.66%) 15 patients (25%) 35 patients (58.3%)
Anthracycline regimen (adjuvant/neoadjuvant)	
Neoadjuvant	48 patients (80%)
Adjuvant	12 patients (20%)
Paclitaxel regimen	
Once weekly	24 patients (40%)
Three-weekly	36 patients (60%)
Maintenance trastuzumab (28 patients)	
Weekly	8 patients (28.5%)
Three-weekly	20 patients (71.5%)

**Table 4.** Dosimetric parameters for both IMRT and 3DCRT plans.

	IMRT (25 patients)	3DCRT (35 patients)
Ipsilateral lung		
V20	17.7%	11.01%
V25	13.5%	9.38%
Dmean	10.2 Gy + 1.18 Gy	5.82 Gy + 2.4 Gy
Heart		
Dmean	3.48 Gy+ 0.82 Gy	1.16 Gy+ 1.16 Gy


V20, Volume of ipsilateral lung receiving 20 Gy; V25, Volume of ipsilateral lung receiving 25 Gy

**Table 5.** Quality of life scores and breast specific domains scores. Values in bold face imply statistical significance.

	Mean (SD)				
Scales\Visit	Baseline (a)	Post CTRT (b)	6 months (c)	*p* value (a) versus (b)	*p* value (a) versus (c)
QLQ-C30 QL PF RF EF CF SF FA NV PA DY SL AP CO DI FI	72.08 (17.00)) 81.78 (17.99) 86.11 (23.20) 78.75 (24.52) 88.05 (20.37) 89.72 (19.91) 30.00 (27.85) 9.44 (13.15) 25.56 (28.03) 10.00 (18.71) 21.11 (30.66) 18.89 (24.06) 20.00 (31.41) 8.89 (16.08) 22.78 (32.18)	70.97 (22.94) 82.67 (18.94) 84.17 (22.00) 82.50 (17.94) 89.17 (18.11) 86.39 (22.86) 28.52 (24.58) 12.22 (18.88) 21.11 (23.14) 11.11 (16.99) 18.89 (24.83) 23.89 (24.62) 15.00 (24.87) 5.56 (13.95) 20.00 (32.59)	76.39 (23.58) 84.67 (15.05) 90.83 (13.87) 81.67 (16.58) 84.72 (17.97) 84.17 (20.22) 23.33 (17.08) 4.72 (14.09) 16.11 (17.62) 7.78 (18.78) 14.44 (21.58) 10.00 (18.71) 9.44 (24.62) 5.56 (17.54) 19.44 (30.25)	0.752 0.808 0.643 0.337 0.753 0.341 0.774 0.301 0.377 0.748 0.658 0.282 0.380 0.203 0.608	0.287 0.309 0.191 0.436 0.328 0.105 0.131 **0.043** **0.026** 0.551 0.171 **0.028** **0.048** 0.307 0.540
QLQ-BR23 BRBI BRSEF BRSEE BRFU BRST BRBS BRAS BRHL	78.05 (27.99) 10.83 (18.37) 13.33 (25.45) 67.78 (41.15) 25.95 (21.27) 10.69 (12.83) 17.96 (17.49) 37.22 (45.14)	80.56 (24.96) 8.05 (13.54) 6.67 (13.44) 74.44 (32.69) 23.97 (20.50) 11.11 (14.69) 14.81 (17.32) 26.11 (38.37)	83.19 (23.94) 11.67 (15.74) 11.67 (20.19) 74.44 (32.69) 11.90 (13.85) 12.92 (15.45) 15.00 (15.01) 10.56 (27.78)	0.583 0.254 0.057 0.367 0.592 0.871 0.353 0.128	0.265 0.274 0.709 0.356 0.9 0.383 0.319 **0.0003**

SD, Standard deviation; PF, Physical functioning; RF, Role functioning; EF, Emotional functioning; CF, Cognitive functioning; SF, Social functioning; FA, Fatigue; NV, nausea-vomiting; PA, Pain; DY, Dyspnoea; SL, Insomnia; AP, Appetite loss; CO, Constipation; DI, Diarrhoea; FI, Financial difficulties; BRBI, Body image; BRSEF, Sexual functioning; BRSEE, Sexual enjoyment; BRFU, Future perspective; BRST, Systemic therapy side-effects; BRBS, Breast symptoms; BRAS, Arm symptoms; BRHL, Hair loss
